# Synthesis of High Valence Silver-Loaded Mesoporous Silica with Strong Antibacterial Properties 

**DOI:** 10.3390/ijerph13010099

**Published:** 2016-01-04

**Authors:** Chun-Chi Chen, Hsin-Hsien Wu, Hsin-Yi Huang, Chen-Wei Liu, Yi-Ning Chen

**Affiliations:** 1 Green Energy and Environment Research Laboratories, Industrial Technology Research Institute, 195 Chung Hsing Road, Chutung, Hsinchu County 31040, Taiwan; chunchi@itri.org.tw (C.-C.C.); cookwu@itri.org.tw (H.-H.W.); cynthia.h@itri.org.tw (H.-Y.H.); Chenwei@itri.org.tw (C.-W.L.); 2 Department of Bioscience Technology, Chung Yuan Christian University, 200 Chung Pei Road, Chung Li District, Taoyuan City 32023, Taiwan

**Keywords:** high valence, silver, SBA-15, antibacterial activity

## Abstract

A simple chemical method was developed for preparing high valence silver (Ag)-loaded mesoporous silica (Ag-ethylenediaminetetraacetic acid (EDTA)-SBA-15), which showed strong antibacterial activity. Ag-EDTA-SBA-15 exhibited stronger and more effective antibacterial activity than commercial Ag nanoparticles did, and it offered high stability of high valence silver in the porous matrix and long-lasting antibacterial activity. The synthesized materials were characterized using Fourier transform infrared spectroscopy, powder X-ray diffraction (XRD), X-ray photoelectron spectroscopy (XPS) analysis, and transmission electron microscopy (TEM). Ag existed in both surface complexation and Ag particles. EDTA anchored within a porous structure chelated Ag ions in higher oxidation states and prevented their agglomeration and oxidation reduction. The XRD results showed that most Ag in the Ag-EDTA-SBA-15 existed in higher oxidation states such as Ag(II) and Ag(III). However, the XPS and TEM results showed that Ag easily reduced in lower oxidation states and agglomerated as Ag particles on the exterior layer of the SBA-15.

## 1. Introduction

Silver (Ag) or its ions have long been known to exhibit strong inhibitory and bactericidal effects as well as possess a broad spectrum of antimicrobial activities [1,2,3]. The antibacterial activity of Ag-containing compounds is currently exploited in myriad applications in various sectors, including hygiene, cosmetics [4,5], antibacterial water filters [6], and medical applications [7]. The bactericidal property of Ag is associated with its valence form [8,9], and studies have found that the higher the valence state is, the stronger and more effective the antibacterial action [10,11]. Recent studies have tested the bactericidal effect of different Ag-based materials and showed that their effect is ordered as follows: AgO > Ag_2_O > Ag [12] and AgNO_3_ > Ag-ZSM-5 > Ag_2_O > commercial Ag-exchanged zeolite (granular) > commercial Ag-exchanged zeolite (pellets) > Ag nanoparticles (NPs) [13]. Although the antibacterial performance of materials with different Ag valence varies in treating various bacteria, the general consensus is that high valence oxides of Ag, such as AgO, possess a strong bactericidal effect. However, because of poor stability, high valence oxides of Ag are rare and far more restricted than those of Ag(I) [14]. Accordingly, silver nitrate (AgNO_3_) and other complexes including silver sulfadiazine, the most widely used topical antibacterial agents, are essentially monovalent Ag antibacterial agents. Designing a stable high valence Ag composite is necessary to achieve effective and long-term antibacterial performance.

Bactericidal activity of simple or composite Ag NPs have been reported in several recent studies [15,16,17,18,19,20,21], in which the oxidation of Ag NPs limits their practical application, as aggregation may cause degradation or even loss of their antibacterial activity [22,23,24]. The use of mesoporous silica as an Ag carrier to overcome this problem has attracted wide attention. For antibacterial applications, Ag-mesoporous silica composites offer many advantages, such as: (1) a higher stability of the Ag ions in the porous matrix (the dispersion of Ag ions within a porous structure prevents their agglomeration and oxidation in air) [25,26,27] and (2) the long-term antibacterial activity of nanocomposites because of the controlled release of Ag ions from the porous matrix [28]. The synthesis of Ag-containing silica has been reported [29,30]. However, to the best of our knowledge, no study has examined the synthesis of mesoporous silica containing high valence Ag. We developed a simple but novel approach based on the oxidation of Ag and stabilization of oxidized high valence Ag(II) through complexation with functionalized mesoporous silica. The mesoporous silica increased the stability, bacterial activity, and durability of the high valence Ag.

SBA-15 is one of the most widely used mesoporous silica materials because of its large surface area and uniform pore structure [31,32,33], which can be modified with functional groups to tailor their properties and achieve specific purposes [33,34,35]. Thus, the development of functionalized SBA-15 as hosts for high valence Ag is interesting and promising. Ag NPs can be embedded into SBA-15, which sometimes is functionalized with specific functional groups such as amine or thiol termini, to form a Ag-silica composite [36,37], but Ag NPs were synthesized by chemical reduction [38,39]. Our goal was to develop a novel approach based on the stabilization of oxidized high valence Ag through complexation with functionalized SBA-15. Song *et al*. synthesized a trivalent Ag complex through the oxidation of monovalent Ag, followed by the stabilization of the oxidized high valence Ag through complexation with a polydiguanide ligand [9]. The immobilization of ethylene- diaminetetraacetic acid (EDTA) on different supporting materials for chelating metal ions has also been reported [40,41,42,43]. Therefore, in this study, we synthesized mesoporous silica SBA-15 with abundant EDTA-chelating groups, and explored its application for stabilizing oxidized high valence Ag within the mesoporous silica.

This is the first paper to report the synthesis of high valence Ag-loaded SBA-15, which demonstrated strong antibacterial activity against Gram-positive and Gram-negative bacteria. The inhibition zones of the high valence Ag composites were considerably larger than those of other Ag-containing materials. The functionalized mesoporous silica (SBA-15) formed high valence Ag composites that demonstrated long-term sterilization (including photostable) activity in ambient conditions. The synthesized materials were characterized using Fourier transform infrared spectroscopy (FTIR), powder X-ray diffraction (XRD), X-ray photoelectron spectroscopy (XPS), and transmission electron microscopy (TEM). The antibacterial activities of these materials were studied by using the disc diffusion assay and minimum concentration (MIC) assay with gram-positive *Staphylococcus aureus* (*S. aureus*) and gram-negative *Escherichia coli* (*E. coli*).

## 2. Experimental Section

### 2.1. Materials

3-Aminopropyltrimethoxysilane (APTES, 95%), absolute ethanol (99.98%), and triblock poly(ethylene glycol)-block-poly(propylene glycol)-block-poly(ethylene glycol) copolymer (Pluronic P123, MW = 5800, EO20-PO70EO20) were purchased from Aldrich (St. Louis, Mo, USA). Tetraethoxysilane (TEOS), hydrochloric acid (HCl, 37%), EDTA, thionyl chloride (SOCl_2_), toluene, dichloromethane (DCM), ether, acetone, sodium carbonate solution, aqueous sodium bicarbonate (NaHCO_3_), nitric acid, sodium hydroxide (NaOH), and lead nitrate were purchased from Sinopharm Chemical Reagent Co., Ltd. (Shanghai, China). All reagents and solvents were of analytical reagent grade and were used as received, except for toluene and DCM, which were distilled just before use. Commercial Ag NPs MNS-Ag-n30 (purity: 98.1%; particle size: 30 nm) were purchased from Uni-Onward Corp. (Taipei, Taiwan). Commercial Ag NPs IONPURE (purity: 99.0%; particle size: 12 nm) were purchased from Ishizuka Glass Corp. (Aichi, Japan). Note that Ag NPs IONPURE also contains SiO_2_ and TiO_2_ nanoparticles. The MNS-Ag-n30 and IONPURE nanoparticles are both inorganic powders, and they remained as stable aqueous dispersions during the test.

### 2.2. Synthesis of High Valence Ag-EDTA Complex

Here, 50 mL of aqueous solution containing 5 g of potassium peroxydisulfate (0.37 M) was held at 85 °C, followed by the addition of 30 mL of NaOH (2.0 M) to maintain the pH value at 5.5. To the resulting solution was added 1.692 g (0.1 M) of AgNO_3_ and 5.07 g of polyvinylpyrrolidone (PVP) as a dispersant and preservative for preventing Ag from aggregation. Then, 3.72 g of EDTA was added to the mixture for maintaining the molar ratio of AgNO_3_ to EDTA at 1:1. After cooling to room temperature, the solid product was recovered by filtration and washed with distilled water. The product was then transferred into a drying box for 24 h, after which the product powder was heated at 50 °C for 3 h.

### 2.3. Synthesis of Functionalized Mesoporous Silica

Typical syntheses of EDTA-SBA-15 were performed according to the protocol in Huang’s study [44], but with the following modifications: Pluronic 123 was stirred with 15 mL of deionized water at 40 °C until it was completely dissolved, followed by the addition of 30 g of 2 M HCl solution and the dropwise addition of 4.4 g of TEOS and 0.248 g of APTES. The mixture was stirred at 40 °C for 20 h and then heated to 100 °C. After cooling to room temperature for 24 h, the product was washed with ethanol for removing surfactant and dried at room temperature. The solid product was denoted as NH_2_-SBA-15. This product was further modified in an anhydrous condition by using DCM as the solvent [45]. After 0.04 mol of EDTA and 100 mL of DCM were mixed in a three-necked flask, 0.04 mol of SOCl_2_ was then slowly added to the mixture through a constant pressure dropping funnel under atmospheric pressure. Immediately after SOCl_2_ was completely added, 1 g of NH_2_-SBA-15 was rapidly added to the mixture. The mixture was stirred at room temperature for 2 h and chemically modified EDTA-SBA-15 was obtained. The solid product was thus separated by filtration, washed in succession with DCM (200 mL), acetone (300 mL), deionized water (1500 mL), NaHCO_3_ (0.1 M/200 mL), deionized water (1500 mL), acetone (300 mL), and deionized water (1500 mL). Finally, the product was dried overnight at 50 °C under vacuum [46].

### 2.4. Synthesis of High Valence Ag-Loaded Mesoporous Silica

The methodology used was essentially that described for the synthesis of high valence Ag-loaded mesoporous silica, except that EDTA-SBA-15, instead of EDTA, was used. The solid product was recovered by filtration and washed with distilled water. The product was then transferred into a drying box for 24 h, after which the product's powder was heated at 50 °C for 3 h.

### 2.5. Instruments and Characterization

FTIR spectra (KBr pellets) in the range of 400–4000 cm^−1^ were taken using a IFS 66 FTIR spectrometer (Bruker, Ettlingen, Germany). The Ag speciation on the surface or bulk of samples was determined by performing XPS and XRD analysis, respectively. XRD patterns were recorded using a PW3040 X-ray diffractometer (Philips, Almelo, The Netherlands) using Cu Kα radiation (k = 1.5406 A). Surface chemistry was analyzed by XPS (ESCALAB 250, VG Scientific, East Grin- stead, UK) with a monochromatic Al Kα (1486.7 eV) X-ray source. Samples were prepared without grinding and directly embedded into the adhesive tape which was placed on a sample disk. The XPS spectra were fitted using PeakFit software and the Pearson VII amp was used as a function for XPS fitting. The product morphology was examined using transmission electron microscopy (TEM; JEM-2100F, 120 kV, JEOL, Tokyo, Japan) and field-emission scanning electron microscopy (FESEM; JSM-6500F, 5 kV, JEOL, Tokyo, Japan). The elemental compositions of the samples were analyzed with energy-dispersive X-ray spectroscopy (EDX) attached to the FESEM instrument. The sample for TEM was prepared by placing a drop of the samples on 200-mesh carbon-coated copper grids. The specific surface areas of the samples were determined by nitrogen adsorption at the temperature of liquid nitrogen with a BET surface area analyzer (Model ASAP 2020, Micrometrics, Norcross, GA, USA).

### 2.6. Bacteria Culture for Antibacterial Activity

Gram-negative *E. coli* (ATCC 10322, Biosafety Level 1) and Gram-positive *S. aureus* (ATCC 10781, Biosafety Level 2) were selected for the antibacterial tests. All microbiological procedures were performed aseptically in a Class II A2 biosafety cabinet (Safzone, Chung Fu, Taiwan). Bacteria culture in the log phase of growth was prepared in tryptic soy broth after 16 h of incubation at 37 °C. The concentration of bacteria culture was determined with optical density measurement at 600 nm (OD600) on a Synergy multidetection microplate reader (BioTek, Winooski, VT, USA). The linear correlation between the density of bacteria and OD600 values indicated 8 × 10^8^ colony forming unit (CFU)/mL at OD600 of 1. Before the tests, the concentrations of bacterial inocula were adjusted to 1.5 × 10^8^ CFU/mL for a disc diffusion assay.

### 2.7. Disc Diffusion Assay

Disc diffusion assay was used to screen the tested materials for their antibacterial efficacy by measuring the inhibition zones around discs with tested materials on Mueller-Hinton agar (MHA) plates inoculated with bacteria. First, the plates (90-mm diameter) were inoculated with *E. coli* or *S. aureus* (1.5 × 10^8^ CFU/mL) by streaking the swabs with bacteria over the entire agar surface to ensure the even distribution of bacteria. Second, 6-mm diameter discs were placed on the surface of plates by using sterile forceps. Next, 5 μL of 100, 10, or 1 mg/mL EDTA-SBA-15 or Ag-EDTA-SBA-15 was added in triplicate to each disc. After the plates were incubated for 18 h at 37 °C, the inhibition zones around the discs were observed and their diameters were measured. Gentamicin, a broad-spectrum antibiotics, and sterile water were used as positive and negative controls, respectively, for antibacterial responses.

### 2.8. Microdilution Method for Minimum Inhibitory Concentration Assay

Microdilution method was used to determine the concentration of tested materials to inhibit 50%, 90%, or 99% of bacteria (MIC_50_, MIC_90_, and MIC_99_) in 96-well microplates. The tested materials were serially two-fold diluted into 10 different concentrations. Each concentration of each tested material had eight wells and each well had 50 μL of the diluted material and 100 μL of 1.5 × 10^8^ CFU/mL *S. aureus* or *E. coli*. Growth media without bacteria, media with bacteria but no tested material, 10 μg of gentamicin and ampicillin were controls for MIC assay. After the incubation at 37 °C for 16 h, the growth of bacteria in each well was determined by the OD600 (Synergy HT, BioTek). MIC_50_, MIC_90_, and MIC_99_ were calculated by the percentage of wells with bacterial growth in the serially diluted concentrations of tested materials.

## 3. Results and Discussion

### 3.1. Formation of High Valence Ag Materials

**Figure 1:**
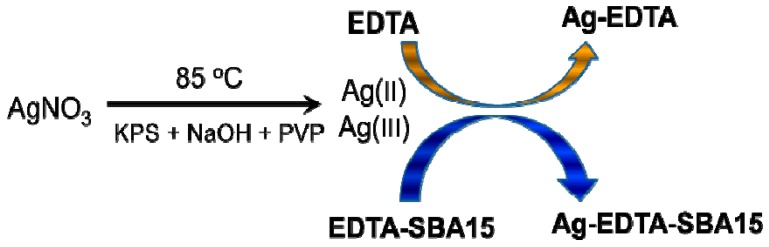
Synthesis of high valence Ag composites.

The procedure used to prepare high valence Ag materials was achieved in three steps (Figure 1). EDTA was impregnated on the surfaces of SBA-15 in the first step. In the second step, Ag^+^ ions were oxidized to high valence Ag. In the presumed Ag(I) complex, some of the *d* electrons of the Ag^+^ ions were compelled to occupy higher energy antibonding orbitals, from which the ions could be removed with peroxydisulfate oxidation to produce a high valence Ag complex. In the third step, EDTA or EDTA-SBA-15 was provided for the complexation and stabilization of high valence Ag.

### 3.2. FTIR Analysis

**Figure 2:**
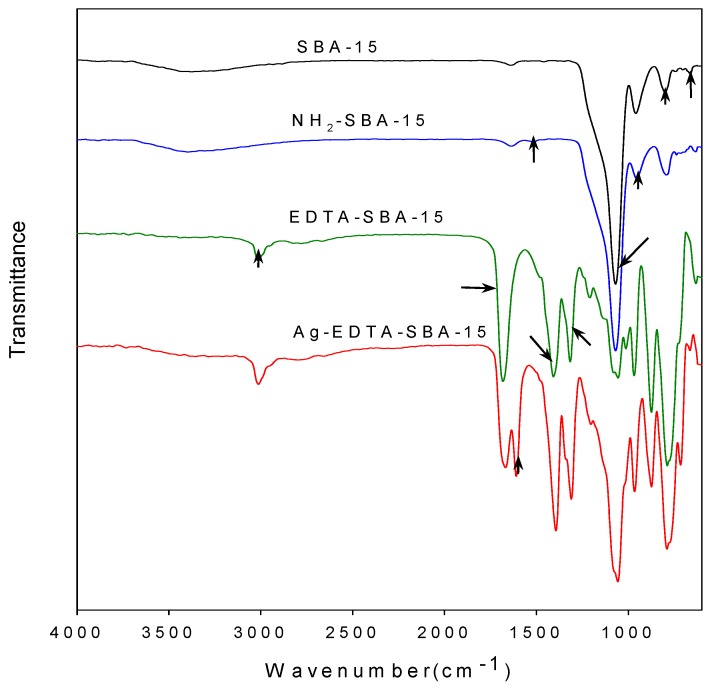
Comparison of FTIR spectra of SBA-15, NH_2_-SBA-15, EDTA-SBA-15, and Ag-EDTA-SBA-15.

The assembly details of the SBA-15 and functionalized SBA-15 were examined using FTIR. The carboxyl groups in the EDTA reacted with SOCl_2_ and changed the carboxyl group into highly reactive acyl chloride [47,48]. The acyl chloride groups in the EDTA then reacted with the amino groups of NH_2_-SBA-15 to form acylamide groups, and EDTA was thus grafted on the matrix surface. The FTIR spectra of prepared materials are presented in Figure 2.

As seen in the figure, bands at 3430, 1638, 1069, 955, 799, and 662 cm^−1^ visible in every sample corresponded to the characteristic vibrations of the silica substrate. The broad band at approximately 3430 cm^−1^ can be attributed to surface silanols and adsorbed water molecules, whose deformational vibrations produced the band near 1638 cm^−1^. The bands at 1069, 799, and 662 cm^−1^ were assigned to Si-O-Si groups [49]. The intensity of Si-OH vibration at 955 cm^−1^ in the NH_2_-SBA-15 was lower than that of the unmodified SBA-15, indicating that most Si-OH bonds on the inner surface of the SBA-15 were occupied because of modification. In addition, the presence of symmetrical -NH_3_^+^ bending at 1510 cm^−1^ proved that aminopropyl groups were successfully grafted on the silica substrate through reactions between APTES and OH groups on the channel wall [50]. Moreover, the elemental analysis results showed that the nitrogen content of the NH_2_-SBA-15 was 1.07 wt %. No other nitrogen source, except for APTES, was introduced during synthesis; in addition, we observed that APTES was well-grafted to SBA-15. Characteristic bands were also observed in the FTIR spectrum of the EDTA-SBA-15 and Ag-EDTA-SBA-15. The peak at 1682 cm^−1^, which was assigned to the C=O stretching vibration of carboxyl group [43], was very intensive for the EDTA-modified sample. The bands at 1000–1400 cm^−1^ were attributed to the C-N stretching and/or C-O stretching vibration, whereas the band at 3000 cm^−1^ was attributed to CH group stretching vibration. The FTIR results confirmed that EDTA-SBA-15 was grafted to the inner surface of SBA-15. After the absorption of Ag ions in the presence of EDTA, a band appeared to be connected with asymmetric stretching vibrations of the C-O band at 1645 cm^−1^.

### 3.3. XPS Analysis

**Figure 3:**
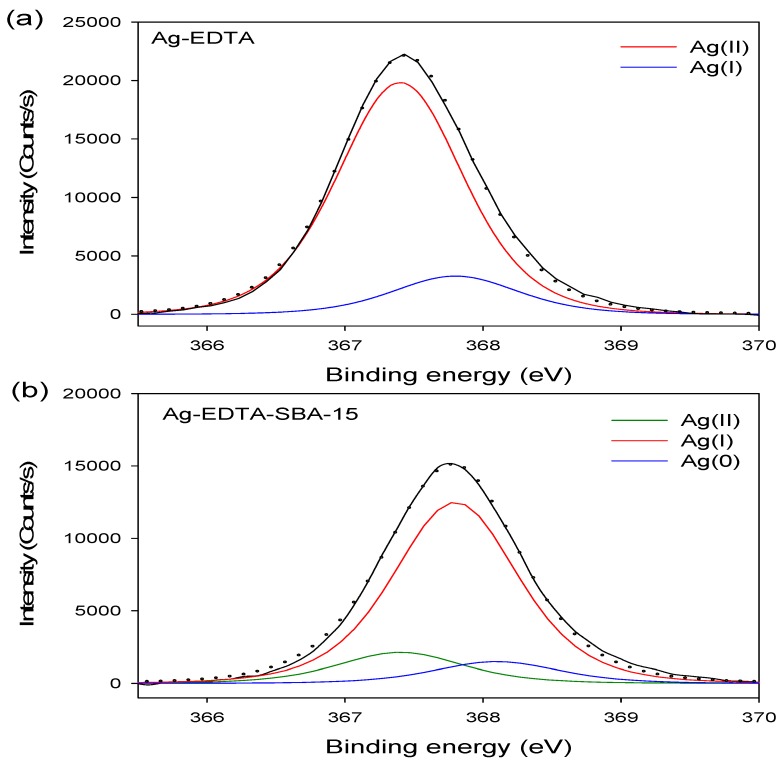
XPS Ag3d spectrum for high valence Ag samples: (**a**) Ag-EDTA and (**b**) Ag-EDTA-SBA-15.

The binding energy of Ag3d_3/2_ and Ag3d_5/2_ in the Ag-EDTA was 373.4 and 367.4 eV, respectively. The Ag3d_5/2_ XPS spectrum of the Ag-EDTA, SBA-15, EDTA-SBA-15, and Ag-EDTA-SBA15 composites are shown in Figure 3. The high-resolution Ag3d_5/2_ spectrum resolved into two individual component peaks (Figure 3a): A small peak at 367.8 eV corresponding to Ag_2_O and a large peak at 367.4 eV corresponding to Ag(II) oxide, as previously reported [51]. Furthermore, 85.9% of Ag compounds existed as AgO; 14.1% existed as Ag_2_O, indicating that through chemical oxidation, the absorbed Ag^+^ ions were oxidized and then directly generated the Ag(II)-EDTA and Ag(I)-EDTA complexes. Most Ag^+^ ions were oxidized to Ag^2+^.

The XPS results showed that doublet characteristics of Ag appeared at 373.8 eV (assigned to Ag3d_3/2_) and 367.8 eV (assigned to Ag3d_5/2_), which only could be demonstrated in the Ag-EDTA-SBA-5 sample. The high-resolution Ag3d_5/2_ spectra of three mesoporous silica materials are shown in Figure 3b. These results confirmed that Ag was grafted on the surface of the EDTA–SBA-15. The peaks at 367.4, 367.8, and 368.1 eV were identified as AgO, Ag_2_O, and Ag, respectively [52]. In addition, 13.2% of element Ag existed as AgO; 77.5% of element Ag existed as Ag_2_O in the silver compounds, whereas 9.3% of element Ag was reduced to metallic Ag. This indicated that element Ag demonstrated mixed valences of Ag(0), Ag(I), and Ag(II) in the mesoporous silica composites.

### 3.4. X-ray Diffraction Analysis

**Figure 4:**
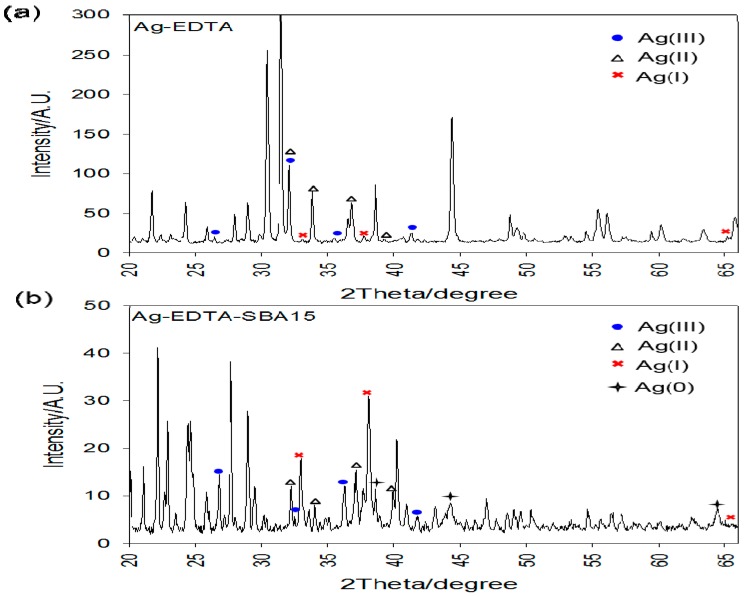
XRD patterns of high valence Ag samples: (**a**) Ag-EDTA and (**b**) Ag-EDTA-SBA-15.

Figure 4a shows wide-angle XRD patterns of the Ag-EDTA composites, and Figure 4b shows the room-temperature wide-angle XRD data of the Ag-EDTA-SBA-15 composites. For the Ag-EDTA sample, the diffraction peaks at 2θ = 26.6°, 32.6°, 36.1°, and 77.1° corresponded to the (1 1 1), (4 2 0), (1 3 1), and (3 3 1) diffraction planes of Ag_2_O_3_, respectively (JCPDS No. 77-1829), indicating the presence of Ag(III). In addition, these peaks appeared along with the characteristic bands of AgO and Ag_2_O (2θ = 32.3°, 37.2°, 34.2°, and 39.4° for AgO and 32.7°, 38.0°, and 65.4° for Ag_2_O). The peak area of Ag_2_O was small than that of Ag_2_O_3_ and AgO. Most of the Ag^+^ ions were oxidized and generated Ag(III) and Ag(II), which were stabilized by complexation with EDTA. For Ag-EDTA-SBA-15 samples, diffraction peaks at 2θ = 38.1°, 44.3°, 64.3°, and 77.3° corresponding to the (1 1 1), (2 0 0), (2 2 0), and (3 1 1) diffraction planes of Ag, respectively, (JCPDS No. 4-0783) appeared along with the characteristic bands of Ag_2_O_3_, AgO, and Ag_2_O. Some Ag^+^ ions oxidized to Ag(III) and Ag(II) and some reduced to Ag(0).

The XPS and XRD results showed that AgO and Ag_2_O were present on the surface of the Ag-EDTA complex, whereas Ag_2_O_3_, AgO, and Ag_2_O were present in the bulk of the substance. These results indicated that high valence Ag is more stable inside the bulk of the substance than it is on the surface of the substance. Similar results were observed for Ag-EDTA-SBA-15 composites, whereas AgO, Ag_2_O, and Ag were found on the surface and Ag_2_O_3_, AgO, Ag_2_O, and Ag were found in the bulk of the substance. Because the high valence Ag phase can be readily photoreduced and is unstable at room temperature compared with Ag_2_O [52,53], in the Ag-EDTA samples, a part of Ag^+^ ions was reduced to metallic Ag, which was not chelated by the anchored EDTA on the surface of the SBA-15. These Ag(0) ions might easily agglomerate as Ag particles.

### 3.5. TEM Analysis

**Figure 5:**
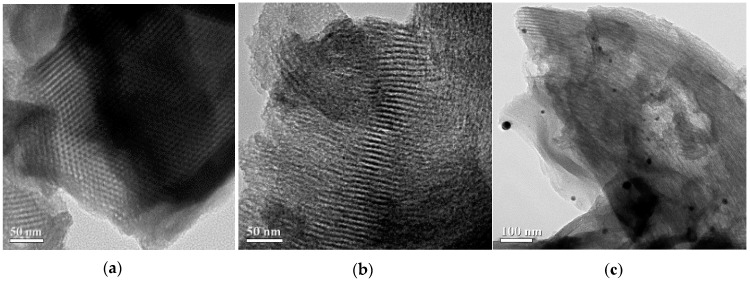
TEM images of (**a**) SBA-15; (**b**) EDTA-SBA-15; and (**c**) Ag-EDTA-SBA-15.

The typical TEM images of mesoporous silica SBA-15 and EDTA-SBA-15 composites are shown in Figure 5a,b, respectively. The micrographs of SBA-15 revealed that the synthesized SBA-15 possessed uniform hexagonal mesostructured pores, with long-range ordering and long channels. The average pore diameter was approximately 9 nm. When EDTA was grafted on the surface of the SBA-15, mesoporous structure was also identified (Figure 5b). In the TEM images of Ag-EDTA-SBA-15 (Figure 5c), spherical Ag particles were dispersed on the surface of the SBA-15. These Ag particles located on the exterior layer were estimated to be approximately 20–100 nm, which is larger than the pore size of SBA-15, as shown by the BET results (Table 1), which revealed only a slight decrease in *S*BET after Ag deposition. This suggests that Ag particle on the SBA-15 surface blocks only a few internal pores, and that the Ag-EDTA-SBA-15 samples maintained a highly mesoporous structure. According to these results and XPS and XRD analysis, this phenomenon may be attributed to the tiny pores in the silica network and the EDTA functional groups grafted on the silica surface. Tiny pores inhibited the growth of the Ag particles within the silica channels and the EDTA functional groups acted as stabilizers to protect high valence Ag^+^ ions from aggregation and anchored them to the silica structure. However, Ag may easily be reduced in lower oxidation states and agglomerate as Ag particles on the exterior layer of SBA-15. This result was consistent with the EDX results, which revealed that high valence Ag-EDTA-SBA-15 was a multiplex process, involving NP formation as well as surface complexation, as discussed in the next section.

**Table 1:** Surface and structural properties of synthesized mesoporous products.

Sample	S_BET_ (m^2^/g)	V_p_ (cm^3^/g)
SBA-15	341	0.48
Ag-EDTA-SBA-15	311	0.45

Vp, pore volume.

### 3.6. EDX Analysis

**Figure 6:**
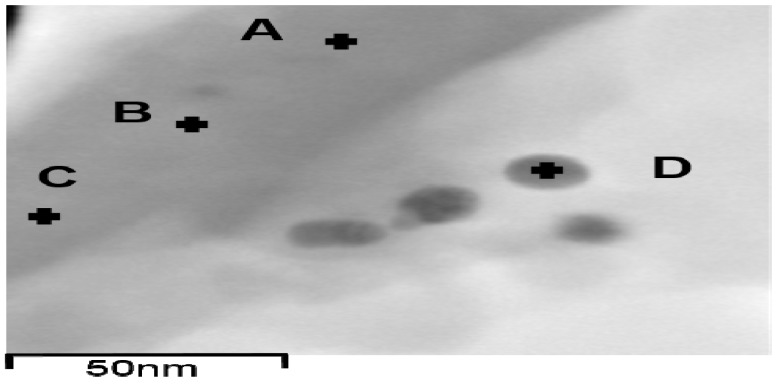
TEM images of EDX position on the surface of Ag-EDTA-SBA-15.

EDX analysis is known for usage of elemental analysis, including metal elements in their metallic or ionized form [54,55,56,57], and was used for probing metallic Ag and ionized Ag in the Ag-EDTA-SBA-15 complex, as shown in Table 2. Most of the elements Ag, Si, and O had atomic percentages of 11.1 wt %–14.4 wt %, 72.3 wt %‒76.0 wt %, and 10.5 wt %‒10.9 wt %, respectively. In Figure 6, position D belongs to Ag NPs, whereas Ag NPs did not appear on the surface of Ag-EDTA-SBA-15 at positions A, B, or C. Element Ag was also observed at positions A, B, and C, which are on the surface of Ag-EDTA-SBA-15 but not on Ag NPs, though the Ag content at these positions was considerably lower than that at position D. This result showed that some high valence Ag was chelated by EDTA on the surface of mesoporous silica as surface complexation, which was not in the particle form. Accordingly, it is surmise that Ag might load on the surface of the silica structure in both the surface-chelated ion and particle forms.

**Table 2:** EDX analysis of the Ag-EDTA-SBA-15 composites.

Label	A	B	C	D
O	10.6	10.9	10.5	0.94
Si	76.0	73.8	72.3	12.8
Ag	11.1	12.9	14.4	86.3

### 3.7. Antibacterial Activity

**Figure 7:**
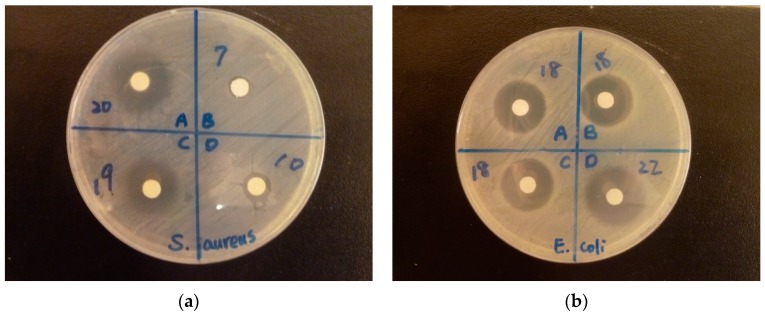
Pictures of the disc diffusion assays: (**a**) *Staphylococcus aureus* (**b**) *Escherichia coli*. The numbers next to the discs are the diameter (mm) of the inhibitory zones.

Disc diffusion assay was performed to compare the antibacterial activity of EDTA, SBA-15, EDTA-SBA-15, Ag-EDTA, Ag-EDTA-SBA-15, and commercial Ag NP products, including IONPURE (Ishizuka) and MNS-Ag-n30 (Uin-onward), against *S. aureus* or *E. coli* (Figure 7).

The final disc diameter of 6 mm was measured on the discs without inhibition zones. was observed around the discs with water in the negative control group, and the final disc diameter was 6 mm. Gentamicin as the positive control had inhibition zones of 24 mm against *E. coli* and 26.3 mm against *S. aureus* on average. The MIC_50_, MIC_90_, and MIC_99_ values of EDTA-SBA-15, Ag-EDTA, and Ag-EDTA-SBA15 were determined by the microdilution method. The results of disc diffusion assay and MICs were listed in Table 3. Although 100 mg/mL of EDTA and EDTA-SBA-15 had large inhibitory zones, no more inhibitory zones were observed after these two materials were diluted to 10 and 1 mg/mL. In addition, no antibacterial activity was detected by the microdilution method for EDTA-SBA-15. Therefore, the critical role of Ag in the antibacterial activity of Ag-EDTA and Ag-EDTA-SBA-15 has been confirmed. The content of Ag in each material depends on the percentage of Ag (% weight) in each material, 98.9% in Ag-EDTA, 19.1% in Ag-EDTA-SBA-15, 1.07% in IONPURE, and 98.7% in MSN-Ag-n30, respectively. Among the tested materials with Ag, Ag-EDTA exhibited the largest inhibition zones, followed by Ag-EDTA-SBA-15, IONPURE, and MNS-Ag-n30. Commercial Ag NPs showed considerably lower antibacterial activity than Ag-EDTA and Ag-EDTA-SBA-15 did. MNS-Ag-n30 almost did not exhibit any antibacterial activity. These results revealed that high valence Ag had stronger and more effective antibacterial action than commercial Ag NPs did. Considering the content of Ag in Ag-EDTA-SBA-15 is 1/5 of those in Ag-EDTA, high valence Ag loaded on mesoporous silica maintained strong antibacterial activity similar to that of the free form of high valence Ag by microdilution method.

**Table 3:** Inhibitory zones (mm) of discs and minimum inhibitory concentrations (MICs) of tested materials against *Escherichia coli* (*E. coli*) and *Staphylococcus aureus* (*S. aureus*).

**Sample**	**Inhibitory Zone (mm)**					
	***E. coli***			***S. aureus***		
	**100 mg/mL**	**10 mg/mL**	**1 mg/mL**	**100 mg/mL**	**10 mg/mL**	**1 mg/mL**
EDTA	9.2 ± 1.9	6 ± 0	6 ± 0	7.7 ± 0.5	6 ± 0	6 ± 0
SBA-15	6 ± 0	6 ± 0	6 ± 0	6 ± 0	6 ± 0	6 ± 0
EDTA-SBA-15	19.5 ± 2.8	6 ± 0	6 ± 0	20.5 ± 0.9	6 ± 0	6 ± 0
Ag-EDTA	23.3 ± 1.2	12.7 ± 2.1	10.5 ± 0.7	23 ± 0	11.3 ± 0.6	8.7 ± 0.6
Ag-EDTA-SBA-15	20.3 ± 0.6	11.7 ± 1.2	9 ± 1	21.3 ± 0.6	11.5 ± 0.5	6.5 ± 0
IONPURE	9.8 ± 0.3	8 ± 0.5	6.5 ± 0	10.5 ± 0.9	6.5 ± 0	6 ± 0
MNS-Ag-n30	7.5 ± 0	6 ± 0	6 ± 0	6.5 ± 0	6 ± 0	6 ± 0
	**Minimum Inhibitory Concentrations (μg/mL)**					
	***E. coli***			***S. aureus***		
	**MIC_50_**	**MIC_90_**	**MIC_99_**	**MIC_50_**	**MIC_90_**	**MIC_99_**
EDTA-SBA-15	(NO Bacterial Inhibition)			(NO Bacterial Inhibition)		
Ag-EDTA	3.4	9.4	16.1	11.4	15.4	16.8
Ag-EDTA-SBA-15	30	39	42	30	47	77

### 3.8. Antibacterial Durability

**Figure 8:**
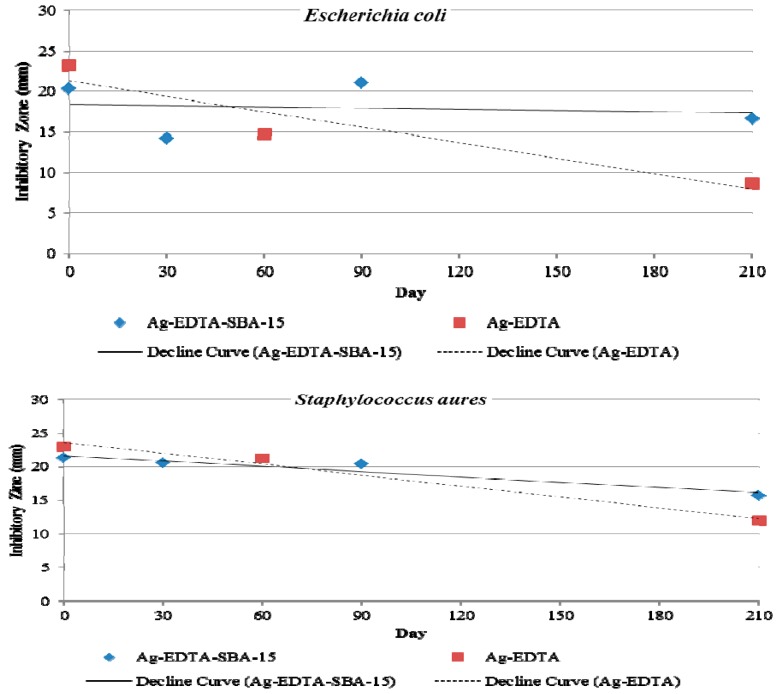
Inhibition zones of Ag-EDTA-SBA-15 and Ag-EDTA against *Escherichia coli* (**top**) and *Staphylococcus aureus* (**bottom**) after storage time of 0, 30, 60, 90, and 210 d.

Long-term antibacterial properties were also evaluated by observing inhibition zones. Figure 8 shows the changes in antibacterial activity against *E. coli* (top) and *S. aureus* (bottom) when Ag-EDTA-SBA-15 and Ag-EDTA were stored in light under ambient conditions for 210 d. At the beginning (0 d), the inhibition zone of the Ag-EDTA was bigger than that of Ag-EDTA-SBA-15 because high valence Ag in the Ag-EDTA had more content. Both the Ag-EDTA and Ag-EDTA-SBA15 maintained antibacterial activity throughout the storage time. After 210 d of storage, the inhibition zone of Ag-EDTA-SBA-15 decreased slightly to 82% (*E. coli*) and 74% (*S. aureus*) of the original zone created by freshly prepared Ag-EDTA-SBA-15. By contrast, after 210 d of storage, the inhibition zone of Ag-EDTA decreased more rapidly and substantially to 37% (*E. coli*) and 52% (*S. aureus*) of the original zone produced by freshly prepared Ag-EDTA. High valence silver is considered to be unstable with highly effective antibacterial performance. The slowly decaying antibacterial activity of Ag-EDTA might result from the photoreduction or agglomeration inducing the reduction of high valence silver when the samples were never protected from light irradiation. The similar condition may also occur on the exterior surface of the Ag-EDTA-SBA-15 samples. Ag-EDTA-SBA-15 was environmentally stable; therefore, its long-term antibacterial effect is expected to be superior to that of Ag-EDTA. These results demonstrated that Ag-mesoporous silica composites indeed offered high stability of high valence Ag in the porous matrix and exhibited long-lasting antibacterial activity.

## 4. Conclusions

**Figure 9:**
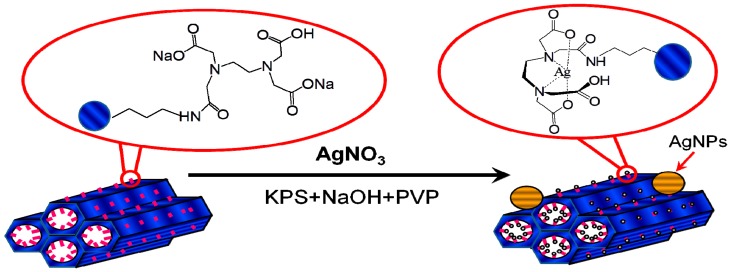
Schematic for the tentative mechanism of high valence Ag formed on the surface of SBA-15.

High valence Ag loaded on mesoporous silica exhibited strong antibacterial activity, which was stronger and more effective than that of commercial Ag NPs. Ag-mesoporous silica composites indeed offered high stability of high valence silver in the porous matrix and yielded long-lasting antibacterial activity. Although the formation mechanism of high valence Ag-SBA-15 composites is not completely clear, the aforementioned results allow us to suggest a tentative process for the formation of Ag-SBA-15 composites, as shown in Figure 9. When EDTA or EDTA-SBA-15 was added, redox reactions between peroxydisulfate and Ag^+^ lead to the formation of high valence Ag. Through XPS, FTIR, XRD, and TEM, the anchored EDTA on the surface of mesoporous silica was discovered to enhance the stability of the high valence Ag complex. XRD showed that most Ag existed in higher oxidation states such as Ag(II) and Ag(III). However, XPS, TEM, and EDX results showed that Ag was easily reduced in lower oxidation states on the exterior layer of SBA-15. Moreover, Ag was present in both surface complexation and NPs. The results in combination showed that EDTA anchored within a porous structure chelated Ag^+^ ions in higher oxidation states and prevented their agglomeration and oxidation in air. The higher stability of high valence Ag^+^ ions in the porous matrix resulted in exceptional bactericidal activity as well as high stability.
